# Vitamin D Status and Efficacy of Vitamin D Supplementation in Atopic Dermatitis: A Systematic Review and Meta-Analysis

**DOI:** 10.3390/nu8120789

**Published:** 2016-12-03

**Authors:** Min Jung Kim, Soo-Nyung Kim, Yang Won Lee, Yong Beom Choe, Kyu Joong Ahn

**Affiliations:** 1 Department of Dermatology, Konkuk University School of Medicine, Seoul 05030, Korea; mjkimmd@kuh.ac.kr (M.J.K.); cyb@kuh.ac.kr (Y.B.C.); kjahn@kuh.ac.kr (K.J.A.); 2 Department of Obstetrics and Gynecology, Konkuk University School of Medicine, Seoul 05030, Korea; snkim@chol.com; 3 Research Institute of Medical Science, Konkuk University, Seoul 05030, Korea

**Keywords:** atopic dermatitis, vitamin D, meta-analysis, systematic review

## Abstract

Recent literature has highlighted the possible role of vitamin D in atopic dermatitis (AD), and that vitamin D supplementation might help to treat AD. This study determined the relationship between vitamin D level and AD, and assessed the efficacy of vitamin D supplementation. We searched the MEDLINE, EMBASE, and Cochrane databases up to May 2015. Observational studies and randomized controlled trials were included based on the available data on the serum 25-hydroxyvitamin D (25(OH)D) level and quantified data available for severity assessed using the Scoring Atopic Dermatitis (SCORAD) index or Eczema Area and Severity Index (EASI) score. Compared with healthy controls, the serum 25(OH)D level was lower in the AD patients of all ages (standardized mean difference = −2.03 ng/mL; 95% confidence interval (CI) = −2.52 to −0.78), and predominantly in the pediatric AD patients (standardized mean difference = −3.03 ng/mL; 95% CI = −4.76 to −1.29). In addition, the SCORAD index and EASI score decreased after vitamin D supplementation (standardized mean difference = −5.85; 95% CI = −7.66 to −4.05). This meta-analysis showed that serum vitamin D level was lower in the AD patients and vitamin D supplementation could be a new therapeutic option for AD.

## 1. Introduction

Atopic dermatitis (AD) is a common and recurring chronic inflammatory disease characterized by pruritus and eczema. It is commonly associated with hypersensitivity to allergens, more frequently with allergic diseases such as allergic rhinitis and asthma [1]. It reportedly affects 5%–20% of children and 1%–3% adults worldwide, and its prevalence is increasing in industrialized countries [2]. The pathophysiology of AD is mainly the result of epidermal barrier defectiveness and immune dysregulation [1,3]. The traditional therapeutic options for AD are antihistamine and immune modulatory agents, including topical/oral corticosteroids and topical/oral calcineurin inhibitors. These classic treatments are focused on reducing skin inflammation [4], but their potential side effects and poor patient adherence indicate the importance of finding new therapeutic options. Recent studies have suggested that vitamin D supplementation may be a safe and effective alternative treatment for AD. A Cochrane review provided evidence for the efficacy of dietary vitamin D supplements as a treatment for AD in 2012 [5]. However, only two studies were reviewed, and owing to their low quality, the review could not produce conclusive evidence for the efficacy of vitamin D supplements in AD treatment.

The present study performed a systematic review and meta-analysis to determine the serum 25-hydroxyvitamin D (25(OH)D) levels in AD patients compared with those in healthy controls. In addition, we reviewed double-blind randomized controlled trials to assess the efficacy of vitamin D supplementation as a treatment for AD by using the Scoring Atopic Dermatitis (SCORAD) index and the Eczema Area and Severity Index (EASI) score.

## 2. Materials and Methods

We conducted this systematic review in accordance with the Preferred Reporting Items for Systematic Reviews and Meta-Analyses statement [6]. We did not register a protocol.

### 2.1. Search Strategy and Data Collection

We performed a systematic review of the MEDLINE, EMBASE, and Cochrane Central Register of Clinical Trials databases up to May 2015 by using the following keywords: (atopic dermatitis OR eczema) AND (vitamin D). The search was limited to human studies. The reference lists of the retrieved articles were reviewed manually.

### 2.2. Study Selection and Data Extraction

The titles and abstracts of the identified articles were checked and independently reviewed by two of the authors (M.K. and Y.L.), with discrepancies resolved by discussion. We included observational studies to determine the association between serum 25(OH)D level and AD. In addition, we included randomized controlled trials to assess the efficacy of vitamin D supplementation as a treatment for AD patients. The following inclusion criteria were applied: (1) presence of AD; (2) data available for serum 25(OH)D level; and (3) quantified data available for AD severity assessed by use of the SCORAD index or the EASI score. We excluded studies that included pregnant women and cord blood samples. We contacted the investigators of the studies by e-mail when necessary to obtain raw or missing data. However, we excluded articles in the meta-analysis that did not contain sufficient data owing the low response rates.

We extracted the following data from the databases: study characteristics (author(s), year of publication, and study design), patient characteristics (age, serum 25(OH)D levels, and SCORAD indices or EASI scores), and vitamin D supplementation (dosages and durations). When the standard deviations (SD) were not available, the probability value was used to assess the SDs.

### 2.3. Quality of Assessment

The quality of each study was assessed by two of the authors by using the nine-star Newcastle–Ottawa Scales (NOS) for observational studies [7]. Independent case–control studies were included, in accordance with the NOS standard procedure. Nine points were given to the study of highest quality, and studies awarded highest quality were considered as having sufficiently high quality in the meta-analysis.

The risk of bias was assessed using the Cochrane Collaboration Risk of Bias Tool for randomized controlled trials [8]. We assessed the risk of bias, random sequence generation, allocation concealment, blinding of participants and personnel, blinding of the outcome assessment, analysis of incomplete outcome data, selective reporting, and other biases. The two authors (M.K. and Y.L.) rated the included articles independently, and the score was finalized by discussion. The results of the assessments were categorized as follows: “yes” for a low risk of bias, “unclear”, and “no” for a high risk of bias.

### 2.4. Statistical Analysis

The mean differences and 95% confidence intervals (CIs) were calculated from the extracted data. We assessed the heterogeneity across the studies using the *I*^2^ statistic, which indicates the percentage of the total variation among studies [9,10]. A funnel plot was constructed to assess the bias based on the standard error [11]. We assessed the interstudy heterogeneity using the *I*^2^ statistic, such that if *I*^2^ was > 50%, the assumption of homogeneity was deemed invalid, and the random effects model was applied; otherwise, the fixed model was used. Data analysis was performed using Review Manager 5.3 (The Cochrane Collaboration, The Nordic Cochrane Centre, Copenhagen, Denmark).

## 3. Results

**Figure 1:**
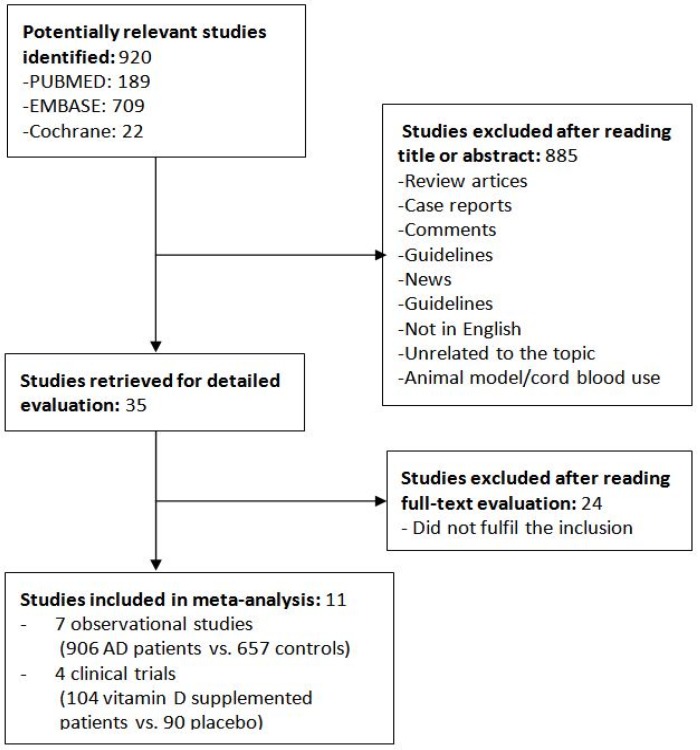
Flow chart of the study selection process. AD: Atopic dermatitis.

**Figure 2:**
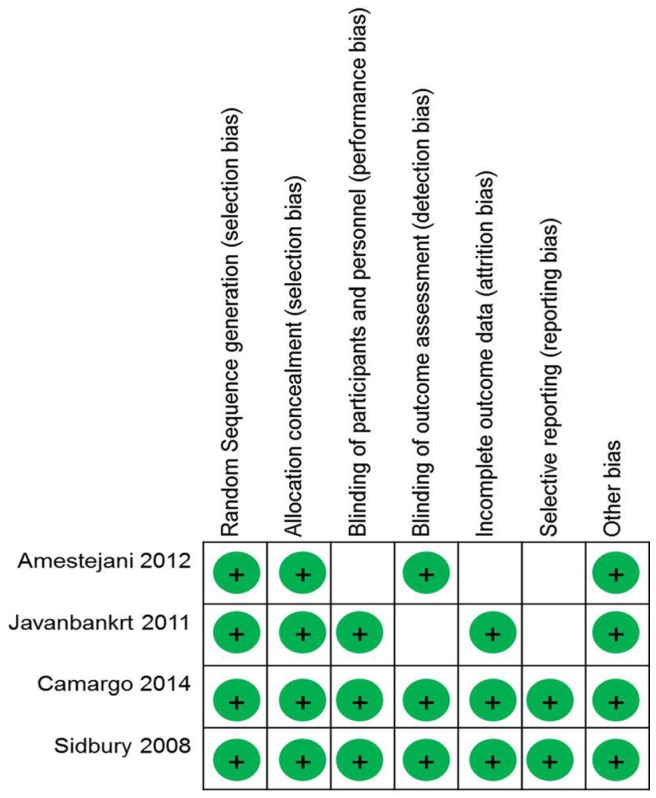
Diagram of the risk of bias of included randomized controlled trials.

Of the 920 articles that were initially retrieved, 35 were selected for full-text review. Eleven of these selected studies were excluded because they did not fulfill the inclusion criteria. Figure 1 shows the flowchart of the studies as assessed through the review process. Table 1 shows the quality of the observation studies by use of the nine-star NOS, and Figure 2 shows the risk of bias of randomized controlled trials.

### 3.1. Comparison of Serum 25(OH)D Levels between AD Patients and Healthy Controls

Seven studies assessed serum 25(OH)D levels in AD patients and in subjects without AD (control group). The characteristics of the included articles are summarized in Table 2. Overall, 986 AD patients and 657 controls were enrolled from all of the included studies. Two studies [14,17] included individuals of all ages, one study [12] included only adults, and four studies [13,15,16,18] included only pediatric patients. The study by Han et al. [17] included individuals of all ages, but the results were categorized into two groups: children and adults.

**Figure 3:**
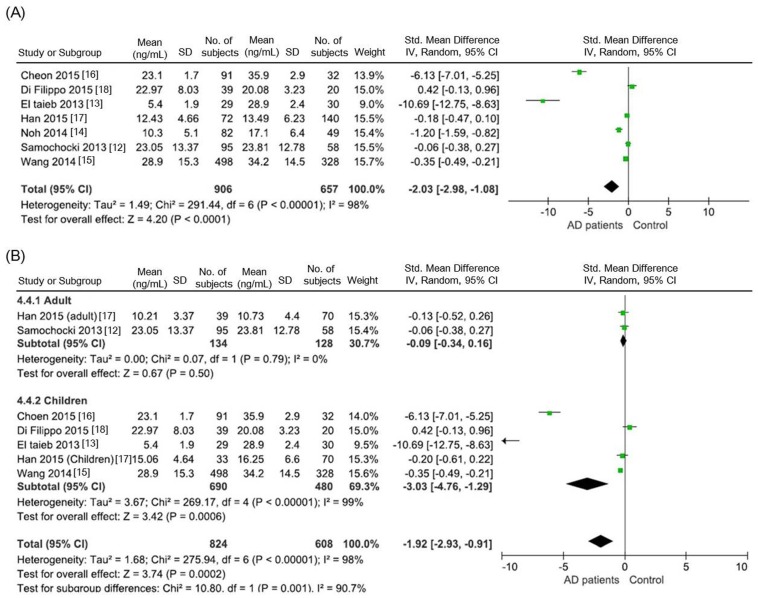
Serum 25(OH)D levels reported in the observational studies. (**A**) Comparison of serum 25(OH)D levels between the AD patients and the controls; (**B**) Subgroup analysis of vitamin D levels: adult AD patients and pediatric AD patients.

Compared with the control group, the AD group had lower serum 25(OH)D levels for individuals of all ages in all the included studies (standardized mean difference = −2.03 ng/mL; 95% CI = −2.98 to −1.08). The included studies were statistically heterogeneous (*I*^2^ = 98%; Figure 3A). In the subgroup analysis, the serum 25(OH)D level was significantly lower in the pediatric AD patients (standardized mean difference = −3.03 ng/mL, 95% CI = −4.76 to −1.29) than in the controls. The serum 25(OH)D level was lower in the adult AD group than in the control group; however, this difference was not statistically significant (standard mean difference = −0.09 ng/mL, 95% CI = −0.34 to 0.16; Figure 3B). Statistical heterogeneity was observed among the included studies. In the subgroup analysis, however, only two studies were conducted in adult AD patients, and the total number of enrolled adult AD patients was small (134 AD patients vs. 128 controls for the adults, and 690 AD patients vs. 657 controls for the children).

### 3.2. Effect of Vitamin D Supplementation in AD Patients

**Figure 4:**
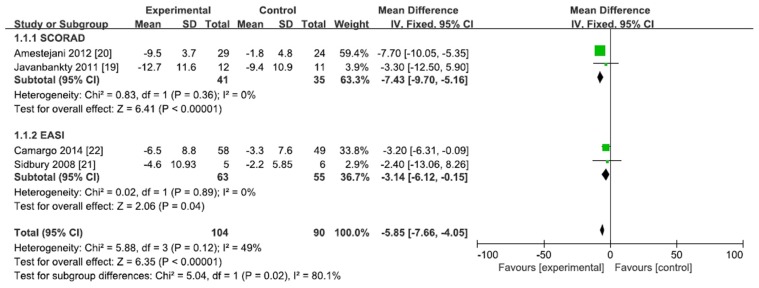
Effect of vitamin D supplementation in the AD patients in the randomized controlled trials.

Four randomized, double-blind, placebo-controlled trials assessed the efficacy of vitamin D supplementation. The characteristics of the included studies are summarized in Table 3. Two studies [19,20] measured the SCORAD indexes, whereas two of the included studies [21,22] assessed the efficacy of vitamin D supplementation by using EASI score. A meta-analysis of four trials showed that SCORAD index and EASI score decreased significantly after vitamin D supplementation (mean difference = −5.85, 95% CI = −7.66 to −4.05). No statistical heterogeneity was observed among the studies (*I*^2^ < 50%; Figure 4).

In addition, we performed a subgroup analysis. SCORAD index was significantly lower in the vitamin D supplement group (mean difference = −7.43, 95% CI = −9.70 to −5.16, *I*^2^ = 0%) and EASI score also showed lower after vitamin D supplementation (mean difference = −3.14, 95% CI = −6.12 to −0.15, *I*^2^ = 0%; Figure 4).

## 4. Discussion

In the present study, we reviewed and comprehensively summarized case–control studies and randomized controlled studies, providing potentially important information. First, serum 25(OH)D level was lower in the AD patients—particularly in the pediatric patients. In addition, this meta-analysis showed that SCORAD index and EASI score were decreased after vitamin D supplementation as a treatment for AD.

Accumulating studies have identified the pathogenesis of AD, including epidermal barrier disruption, immunologic dysfunction, and personal susceptibility. Based on genetic susceptibility (such as loss of filaggrin gene), the AD patients were found to have a defective skin barrier and dysregulation of the innate immune system, which results in failure of immunologic responses to allergens and microbial pathogens [23,24]. The association between vitamin D deficiency and AD is not clear, but several studies have suggested possible roles of vitamin D in AD. A previous study demonstrated that antimicrobial peptides such as cathelicidin and β-defensin increased after vitamin D supplementation in vitro [25]. Liu et al. [26] also found antimicrobial response triggered by the Toll-like receptor and vitamin D-mediated immunity. Clinical trials also showed that vitamin D supplementation promotes cathelicidin production and induces LL-37 expression. Thus, vitamin D promotes antimicrobial activity, with a lower vitamin D level thereby reducing antimicrobial activity and external tolerability to pathogens, making it key in the pathogenesis of AD [27].

Serum 25(OH)D level was lower in the AD patients than in the healthy controls across all ages (standardized mean difference = −2.03 ng/mL). Several studies were performed to investigate the association between vitamin D deficiency and AD. However, the association between 25(OH)D and AD is controversial, particularly in adult patients. A large-scale cohort study involving Korean adult patients found that lower serum 25(OH)D levels were associated with AD [28]. On the contrary, a recent Danish cohort study found that serum 25(OH)D level was not associated with AD and other allergic diseases in adults [29]. The above-described results prompted us to perform a sub-group analysis after dividing AD patients into an adult group and a pediatric group. We found that serum 25(OH)D level was markedly lower in the pediatric patients. Pediatric AD patients may have an increased risk of allergen penetration through the skin, and most allergies are initiated in childhood. Therefore, we believe that a low vitamin D level could worsen AD, especially in pediatric patients. In adult AD patients, the serum 25(OH)D level was lower than the control group, but this difference was not statistically significant. Our results should be interpreted with caution, because the total number of adult AD patients enrolled in our study was small. Further larger and well-controlled observational studies are required in the future to consolidate the association between serum 25(OH)D and AD.

The effect of vitamin D supplementation should be considered from two aspects: AD incidence and modification of AD severity [21]. We focused on AD severity, which is associated with disease modification. The present study included four randomized double-blind clinical trials and found that vitamin D supplementation decreased the SCORAD index and EASI score, and that vitamin D supplementation benefits AD patients. However, Norizoe et al. [30] gave vitamin D supplements to mothers during lactation, which did not improve the AD status of the infants. These authors suggested that vitamin D supplementation in infants during breast feeding could increase the risk of food allergy later in life, and supplementation during lactation could have a negative effect on the development of the Th1 and Th2 immune balance [31]. Therefore, vitamin D supplementation should be provided carefully whilst considering the age of the patient, and further studies will be needed.

Two of the included clinical trials (by Sidbury et al. [21] and Camargo et al. [22]) enrolled pediatric patients who had a history of AD worsening in the winter, and both studies showed that EASI score was decreased after vitamin D supplementation. These authors explained that the lower sun exposure in the winter leads to vitamin D deficiency, and lower vitamin D levels could worsen AD. Similarly, a recent study reported that birth in winter season may be associated with AD, which suggested that inadequate sunlight exposure (associated with lower vitamin D status) increased the risk of AD [32]. Accumulating data have proven that phototherapy is the most efficacious, well-tolerated treatment option for AD. Ultraviolet B radiation suppresses the expressions of pro-inflammatory cytokines such as interleukin (IL)-12, IL-2, and interferon-γ, and stimulates IL-10 production via keratinocytes, thereby reducing expression levels of pro-inflammatory cytokines and suppressing the growth of natural killer cells [33,34]. Thus, vitamin D supplementation can compensate for the reduced ultraviolet exposure and alleviate AD severity. From these data, we assume that vitamin D supplementation is effective in AD patients, and that the characteristics of their condition—including seasonal variation of severity—should be considered.

The results of this study should be interpreted with caution, because of the several limitations of our meta-analysis. First, information about basal 25(OH)D levels and changes in 25(OH)D level after vitamin D supplementation was lacking. Heaney et al. [35] suggested a guideline for clinical studies of nutrient effects. The author emphasized the measurement of basal levels of nutrients, and that when performing meta-analysis, all the included studies should have similar basal nutrient levels. Owing to the inadequate information from the included articles, we could not provide the exact status of 25(OH)D level. Javanbakht et al. [19] did not report basal 25(OH)D levels in detail, but they reported the prevalence of vitamin D deficiency, which was 72.7% in the placebo group and 81.8% in the vitamin D supplemented group. Amestejani et al. [20] also measured 25(OH)D level and reported a mean 25(OH)D level of 9.8 ng/mL before vitamin D supplementation, which was categorized as vitamin D-deficient status. Studies performed by Camargo et al. [22] and Sidbury et al. [21] did not provide data on 25(OH)D levels. However, these studies included the AD patients who had a history of worsening in the winter season, and the authors assumed that the worsening in the winter season was associated with vitamin D deficiency. Thus, we assumed that the included AD patients in this meta-analysis probably had vitamin D deficiency and similar 25(OH)D levels. Second, we did not adjust for potential confounding factors of the serum 25(OH)D level in the selected populations, such as altitude, latitude, sun exposure, seasonal variation, level of outdoor activities, and dietary vitamin D intake [36]. Third, significant interstudy heterogeneity was present, which can be partially attributed to the confounding factors. The study included uncontrollable factors of AD treatment, such as usage of topical steroid and confounding factors as mentioned earlier. Finally, this meta-analysis included a small number of randomized clinical trials in the assessments of the efficacy of vitamin D supplementation. However, we thought a meta-analysis would still be worthwhile to determine the overall association between serum 25(OH)D and SCORAD index. Further larger studies that include a subgroup analysis of infant, pediatric, and adult AD patients are required to clarify the benefits of vitamin D supplementation.

In conclusion, this meta-analysis summarized the evidence for the role of vitamin D in AD patients. Serum 25(OH)D level was lower in the AD patients than in the controls, and the subgroup analysis showed that the difference in serum 25(OH)D level was significant in the pediatric patients. In addition, in comparison with a placebo group, vitamin D supplementation decreased AD severity and improved the symptoms and clinical signs of AD. However, the specific mechanisms underlying the role of vitamin D in this relationship are still unclear. Further studies are required to clarify the molecular pathways and mechanisms underlying the effects of vitamin D, with large-scale clinical trials needed to assess the effect of vitamin D treatment on AD outcomes.

## Figures and Tables

**Table 1:** Assessment of the quality of the included observation studies by using the Newcastle–Ottawa scale.

Study	Year	Criterion Scores		
		Selection	Comparability	Exposure
Samochocki et al. [12]	2013	★★★★	★★	★
El Taieb et al. [13]	2013	★★★★	★★	★
Noh et al. [14]	2014	★★★★	★★	★
Wang et al. [15]	2014	★★★	★★	★
Cheon et al. [16]	2015	★★★		★
Han et al. [17]	2015	★★	★★	★
Di Filippo et al. [18]	2015	★★★	★★	★

**Table 2:** Summary of the characteristics of the included studies for the comparison of serum 25-hydroxyvitamin D (25(OH)D) levels between AD patients and healthy controls.

Study	Year	Study Population	Study Size	Location
Samochocki et al. [12]	2013	Adults aged 18–50 years (mean age: 29.9 years)	95 cases, 58 control subjects	Poland
El Taieb et al. [13]	2013	Children aged 2–12 years (mean age, AD group: 6.2 years, control group: 6.5 years)	29 cases, 30 control subjects	Egypt
Noh et al. [14]	2014	All ages (mean age, AD group: 20.8 years, control group: 29.5 years)	82 cases, 49 control subjects	Korea
Wang et al. [15]	2014	Children (mean age: 15.5 years, control group: 12.3 years)	498 cases, 328 control subjects	Hong Kong
Cheon et al. [16]	2015	Children (median age, AD group: 6 years, control group: 6 years)	91 cases, 32 control subjects	Korea
Han et al. [17]	2015	All ages (adult group aged 18–51 years, pediatric group aged 12 months–16 years)	72 cases, 140 control subjects	Korea
Di Filippo et al. [18]	2015	Children (mean age, AD and control groups: 4 years)	39 cases, 20 control subjects	Italy

**Table 3:** Summary of the characteristics of randomized clinical trials investigating the efficacy of vitamin D supplementation.

Study	Year	Study Design	Study Population	Study Size	Dose and Frequency	Supplemented Vitamin D	Duration	Location	AD Severity Assessment	Changes in Severity Index (Experimental, Control)	SCORAD or EASI Index (Experimental (before→after), Control (before→after))
Javanbakht et al. [19]	2011	Randomized double-blind placebo controlled	All aged, 13–45 years	12 cases, 11 placebo	1600 IU, daily	Cholecalciferol (Vitamin D3)	60 days	Iran, Tehran	SCORAD *	−12.7 ± 11.6, −9.4 ± 10.9	36.0 ± 3.7→23.3 ± 2.8, 31.7 ± 3.5→22.3 ± 3.0
Amestejani et al. [20]	2012	Randomized double-blind placebo controlled	>14 years	29 cases, 24 placebo	1600 IU, NR	Cholecalciferol (Vitamin D3)	2 months	Iran	SCORAD	−9.5 ± 3.7, −1.8 ± 4.8	24.8 ± 4.1→15.3 ± 3.1, 25.3 ± 5.2→23.46 ± 4.2
Sidbury et al. [21]	2008	Randomized double-blind placebo controlled	Children, median: 7 years	5 cases, 6 placebo	1000 IU, NR	Ergocalciferol (Vitamin D2)	1 month	USA	EASI *	−4.6 ± NR, −2.2 ± NR	NR *
Camargo et al. [22]	2014	Randomized double-blind placebo controlled	Children, mean: 9 years	58 cases, 49 placebo	1000 IU, daily	Cholecalciferol (Vitamin D3)	1 month	Ulaanbaatar, Mongolia	EASI	−6.5 ± 8.8, −3.3 ± 7.6	NR

* SCORAD: Scoring Atopic Dermatitis, EASI: Eczema Area and Severity Index, NR: not reported.
